# Hot Water Epilepsy in a Pregnant Woman: A Case Report

**DOI:** 10.1155/2010/134578

**Published:** 2010-12-13

**Authors:** Aysel Milanlıoğlu, Temel Tombul, Refah Sayın

**Affiliations:** ^1^Department of Neurology, Bitlis State Hospital, 13100 Bitlis, Turkey; ^2^Department of Neurology, Faculty of Medicine, Yüzüncü Yıl University, 65080 Van, Turkey

## Abstract

Hot water epilepsy is a unique form of reflex epilepsy precipitated by the stimulus of bathing with hot water poured over the head. It is mostly seen in infants and children, with a predominance in males. Unlikely, we present a 32-year-old pregnancy woman with the incipient of reflex seizures triggered by pouring hot water over the head while having a bath during the gestation period and treated successfully with carbamazepine 400 mg/day therapy. Hot water epilepsy is known as a benign and self-limited reflex epilepsy, by firstly avoiding hot water or long showers and secondly using intermittent benzodiazepines or conventional antiepileptic drugs, may be sufficient to be seizure-free.

## 1. Introduction

Hot water epilepsy (HWE) is a rarely seen, benign form of reflex epilepsy which is precipitated by the stimulus of bathing with hot water poured over the head. It is considered to be a geographically specific epileptic syndrome since it mainly occurs in India.

Almost all cases of HWE are seen in healthy children, with the cases more frequent among male than female patients [1].

Interestingly, we report a 32-year-old pregnant woman with the onset of reflex seizures triggered by pouring hot water over the head while having a bath.

## 2. Case Report

A 32-year-old, three-month pregnant woman came to our outpatient clinic with the complaint of incipient seizures while having a bath by pouring hot water over the head since two months earlier. She had auras preceding her seizures. These auras were associated with feeling a epigastric sensation, staring, oral automatism, and followed by loss of consciousness. Postictal state was characterised by a severe throbbing headache and drowsiness. Seizures occurred twice a month and always during bathing. Till the admission, she had four similar seizures. She had no spontaneous seizures before the onset of her reflex seizures. There were no family history of epilepsy and no past history of febrile convulsions, mental retardation, birth anoxia, or head trauma. Physical and neurological examinations were normal. Complete blood count, blood biochemistry, electrocardiography, interictal electroencephalography (EEG), and magnetic resonance imaging also revealed normal findings.

Avoiding the seizures, short-lasting bathing with lukewarm water instead of hot water was recommended. One month followup, her seizures did not stop during regular bath. Hence, she was put on carbamazepine 400 mg/day and completely remained seizure-free.

## 3. Discussion

HWE is a reflex epilepsy in which the seizures are provoked by contact with hot water over the head [2].

A large number of patients with HWE have been reported from India.There have been some case reports from all round the world, such as Turkey.

 Traditionally, Turkish people bathe by sitting and pouring hot water over the heads with a bowl. The temperature of water varies between 40 and 50°C, and water poured on head can cause seizure. Similarly, the main precipitating factors for seizures in our case were bathing with hot water and pouring water over the head. It seems that traditional bathing habits are very important in this type of epilepsy [3].

To date, the pathophysiologic mechanism of HWE is not known clearly but apparently the thermoregulatory system, which is extremely sensitive to the rapid rise in temperature, seems to be detrimental [4].

HWE is mostly seen in the first decade of life, with cases more frequent among male than females (70%). However, some features of our patient such as the initiation age, gender, and additional existence of gestation were different from the literature. Because of this, our case is an unusual presentation of HWE.

The pattern of epileptic seizure which is seen in HWE, consists of 67% complex partial seizure and 33% generalized tonic-clonic seizure. Interictal EEG studies are usually normal like in our case whereas ictal EEG usually shows focal epileptic activities and paroxysmal discharges characterized by secondary generalization [5]. İctal recording was not done for our patient because of the difficulty and disability in provoking such a reflex seizure.

HWE is known as a benign and self-limited reflex epilepsy, only by avoiding hot water or long showers it may be sufficient to be seizure-free. However, approximately one-third of patients with HWE continue to have seizures even during regular baths. In these patients, carbamazepine, valproic acid, or intermittent oral prophylaxis with benzodiazepines before bathing might be preferred. In our case, we used carbamazepine, one of the conventional antiepileptic drugs, and achieved sufficient seizure control. Since seizures show a tendency to decrease, withdrawal of medication should be carefully undertaken only after several years [6].

Finally, HWE has usually favorable prognosis by firstly avoiding hot water and secondly using intermittent benzodiazepines or antiepileptic drugs.
